# A Comprehensive Review of Bioactive Compounds from Lactic Acid Bacteria: Potential Functions as Functional Food in Dietetics and the Food Industry

**DOI:** 10.3390/foods12152850

**Published:** 2023-07-27

**Authors:** Bibi Nabihah Abdul Hakim, Ng Jia Xuan, Siti Nur Hazwani Oslan

**Affiliations:** 1 Faculty of Food Science and Nutrition, Universiti Malaysia Sabah, Jalan UMS, Kota Kinabalu 88400, Sabah, Malaysia; bibinabihah@ums.edu.my (B.N.A.H.); ng_jia_bn20@iluv.ums.edu.my (N.J.X.); 2 Innovative Food Processing and Ingredients Research Group, Faculty of Food Science and Nutrition, Universiti Malaysia Sabah, Jalan UMS, Kota Kinabalu 88400, Sabah, Malaysia

**Keywords:** lactic acid bacteria, fermentation, bioactive compound, functional properties, application of LAB

## Abstract

Lactic acid bacteria (LAB) are beneficial microbes known for their health-promoting properties. LAB are well known for their ability to produce substantial amounts of bioactive compounds during fermentation. Peptides, exopolysaccharides (EPS), bacteriocins, some amylase, protease, lipase enzymes, and lactic acid are the most important bioactive compounds generated by LAB activity during fermentation. Additionally, the product produced by LAB is dependent on the type of fermentation used. LAB derived from the genera *Lactobacillus* and *Enterococcus* are the most popular probiotics at present. Consuming fermented foods has been previously connected to a number of health-promoting benefits such as antibacterial activity and immune system modulation. Furthermore, functional food implementations lead to the application of LAB in therapeutic nutrition such as prebiotic, immunomodulatory, antioxidant, anti-tumor, blood glucose lowering actions. Understanding the characteristics of LAB in diverse sources and its potential as a functional food is crucial for therapeutic applications. This review presents an overview of functional food knowledge regarding interactions between LAB isolated from dairy products (dairy LAB) and fermented foods, as well as the prospect of functioning LAB in human health. Finally, the health advantages of LAB bioactive compounds are emphasized.

## 1. Introduction

Over time, the purpose of food has evolved beyond mere taste and nutrition, now serving as a powerful means to enhance human health through added functionality. Diet plays a crucial role in overall human health, serving as a frontline defense against numerous diseases [1]. As the interest in the relationship between food and health continues to rise, the demand for functional foods is also increasing. While there is no universally accepted definition, functional foods are generally described as food products that offer various health benefits when incorporated into one’s diet. Functional foods can be classified into four main categories according to their definition: conventional foods, modified foods, foods designed for special dietary needs, and medicinal foods [2]. There is a growing interest in characterizing and incorporating bioactive constituents into foods in order to satisfy medically defined criteria and nourish populations.

A recent strategy that has gained traction is the use of fermentates, which are powdered formulations formed from fermentation reactions. Fermentates can be made up of either the bacteria that are fermenting or the metabolites and bioactive compounds that are excreted in the fermentation broth. Milk and dairy products are consumed by more than six billion people worldwide, as they are a food group with a wide variety in terms of taste, texture, and nutritional value [3]. Milk is enriched with useful components, such as minerals and vitamins [4]. In particular, fermented dairy products are a good source of different species of live lactic acid bacteria (LAB) [5].

Peptides, exopolysaccharides (EPS), bacteriocins, some amylase, protease, lipase enzymes, and lactic acid are the major bioactive molecules produced by LAB activity during fermentation [6]. However, not all LAB strains can generate these compounds. The health benefits of LAB have made them a popular ingredient in therapeutic nutrition. One of the most common ways that LAB is used in therapeutic nutrition is in the form of probiotic supplements [7]. Fermented dairy products may show their health-promoting effects due to the influences of microbial metabolites (biogenic or bioactive effect) formed during the fermentation process, as well as the probiotic effects of certain LAB strains isolated from their composition [8]. Many studies have stated that the consumption of probiotic-containing dairy products such as yogurt, cultured fermented milk, and kefir has been linked to a variety of health benefits, such as cholesterol metabolism and angiotensin-converting enzyme (ACE) inhibition, antimicrobial activity, tumor suppression, faster wound healing, and immune system modulation [9,10]. Moreover, the consumption of probiotics balances the gut and urinary tract microbiome by promoting the growth of beneficial bacteria that outcompete pathogens for food and binding sites and locally generate antimicrobial metabolites. As a side benefit, probiotics help the mucosal barrier function by influencing the host immune system [11].

This article summarizes the complex relationships between dairy LAB and human health, and suggests an innovative approach to describing and incorporating bioactive compounds into foods in order to serve as a crucial functional food. These bioactive compounds have been studied for their potential in treating food intolerance, gastrointestinal complications, diabetes mellitus, inflammatory bowel disease, liver disease, and cancer, and the article emphasizes the fermentation and/or probiotic potentials of dairy LAB.

### 1.1. Characteristics of LAB

Lactic acid bacteria (LAB) play a pivotal role in the food processing industry, serving as a vital group of bacteria with substantial significance. Most of these microorganisms are “generally recognized as safe” (GRAS) because they are nonpathogenic, useful in technological and industrial processes, acid and bile tolerant, and are able to produce antimicrobial substances; they have also been consumed by people all over the world for a long time in dairy products [12]. LAB is a well-known class of microorganisms used in the food industry due to their wide range of phylogenetic and functional diversity. LAB are defined as a taxonomic order of bacteria that is both phylogenetically and functionally diversified [13]. Lactic acid bacteria from the genera *Lactobacillus* and *Enterococcus* are currently the most popular probiotics. These LAB strains include *L. acidophilus*, *L. fermentum*, *L. casei*, *L. reuteri*, *L. rhamnosus*, *L. helveticus*, *L. lactis*, *L. crispatus*, *L. gasseri*, *L. plantarum*, and *E. faecalis* [14].

In the last 20 years, *Lactobacillus* has emerged as the preeminent nomenclature for probiotics, gaining widespread popularity in scientific discourse and research. Therefore, it is important to approach the probiotic potential of LAB genera with caution and subject them to individual assessment and scrutiny to determine their suitability as probiotics. Some LAB genera are probiotics, although scientists disagree. The most researched strains of probiotic LAB include *Lactobacillus acidophilus* NCFM, *Lactobacillus acidophilus* LA-5, *Lactobacillus* casei DN-114 001, *Lacticaseibacillus casei* strain Shirota, *Lacticaseibacillus casei* Zhang, and *Lactobacillus rhamnosus* GG (ATCC53103) [11]. Recently, novel probiotic LAB such as *Limosilactobacillus reuteri* and *Lactobacillus johnsonii* have been employed in the production of functional dairy products [15]. More research is required to screen and unravel the probiotic potentials of novel LAB strains with unique favorable health effects on both humans and animals, and that are of scientific and industrial value, because probiotic traits and features are strain-specific [16]. Moreover, the attributes of probiotics can include various characteristics, such as hemolytic activity and antibiotic resistance [17,18]. However, it is important to carefully evaluate these attributes, as excessive hemolytic activity can be harmful and antibiotic resistance may have implications for antibiotic effectiveness. Other features include the ability to adhere, the capacity to inhibit or eliminate harmful microbes, to auto- and co-aggregate, and to be harmless to animals. Li et al. [19] demonstrated that all five isolates had significant adhesion potential, extraordinary aggregation capacity, and antibacterial properties.

### 1.2. Source of LAB in Dairy Products

Milk and other dairy products are generally thought to be the principal dietary sources for LAB. Throughout the world, people drink either fresh or fermented cow and goat milk. Table 1 has shown source of LAB in dairy and non-dairy products. High numbers of LAB as beneficial bacteria in milk suggest a source for biological materials with great public health value and extensive applicability in the dairy sector [20]. According to Agagunduz et al. [11], the milk-based sources employed (kind of animal, diet, age, length of the lactation period, etc.) and food processing techniques (temperature, storage conditions, etc.) are the two primary elements that determine the nutritional value of dairy products. The beneficial health effects of fermented milk and dairy products are mostly attributed to the presence of LAB, which can be naturally found in some dairy products. The most common dairy products that contain LAB are fermented milk, yogurt, cheese, and other milk products [21]. They may be included as a starter culture or occasionally as novel ingredients or additives for the purpose of boosting the functionality of the product, and their ability to increase the nutritional value of fermented milk products [22]. Due to their long history of usage in food and milk fermentation, LAB starter cultures are now classified as GRAS [23].

While there is no definitive cell count number that can ensure the health effects of the probiotic strain in a food product, it has been shown that at least 10^6^–10^8^ cfu/g is adequate to benefit from the advantageous effects of probiotics [24]. This very clearly demonstrates that just because a culture that has the potential to show probiotic potential is present in a product does not necessarily mean that the product itself will have probiotic properties. Probiotics are only effective against certain strains of bacteria; thus, even various strains of the same species might have wildly diverse effects on the host. As a result, it is indicated that more research is needed to understand the probiotic potential of new LAB strains as well as well-known dairy product starting cultures [25].

**Table 1:** Source of LAB in dairy and non-dairy products.

LAB Source	Family	Genus	Gram	Shape	Acid- Resistant	Respiration	References
Dairy Product	*Lactobacillaceae*	*Lactobacillus*	+	Rod shaped	Changeable	Facultative anaerobic	[26]
		*Pediococcus*	+	Spherical shaped	High acid resistant	Facultative anaerobic	[27]
	*Steptococcaceae*	*Streptococcus*	+	Coccoid shaped	Low acid resistant	Facultative anaerobic	[28]
		*Lactococcus*	+	Coccoid	Changeable	Facultative anaerobic	[29]
	*Leuconostocaecae*	*Leuconostoc*	+	Spherical, oval shaped	Changeable	Facultative anaerobic	[30]
	*Bifidobacteriaceae*	*Bifidobacterium*	+	Rod-branch-shaped	High acid resistant	Anaerobic	[31]
	*Enterococcaceae*	*Enterococcus*	+	Coccoid shape	Moderate acid resistant	Facultative anaerobic	[31]
	*Propionibacteriaceae*	*Propionibacterium*	+	Rod shaped	Low acid resistant	Anaerobic	[30]
Non-diary	*Aerococcaceae*	*Aerococcus*	+	Coccoid shaped	Low acid resistant	Facultative anaerobic	[32]
	*Carbobacteriaceae*	*Carnobacterium*	+	Rod shaped	Not available	Facultative anaerobic	[33]
	*Leuconostocaecae*	*Oenococcus*	+	Spherical shaped	Changeable	Facultative anaerobic	[34]
		*Weissella*	+	Coccoid or rod shaped	Changeable	Facultative anaerobic	[35]
		*Fructobacillus*	+	Elongated and slightly cylindrical shaped	Not available	Facultative anaerobic	[36]
	*Enterococcaceae*	*Tetragenococcus*	+	Coccoid shaped	Changeable	Facultative anaerobic	[37]
		*Vagococcus*	+	Coccoid shaped	Changeable	Facultative anaerobic	[38]

### 1.3. Source of LAB in Fermented Food

#### Fermented Food

Fermented foods have become an important branch of the food industry as these foods are abundant sources of potential beneficial microbes that extend the shelf life and increase the nutritional variety and organoleptic properties of the food [39]. Historically, fermented food has been consumed as a staple food since the development of human civilizations. The functional microorganisms naturally present in fermented food offer unique properties to the consumer, including antimicrobial and antioxidant properties and bioactive compound production [40]. Certain strains of probiotics, such as *Lactobacillus*, *Leuconostoc*, and *Enterococcus*, have the ability to thrive and remain viable throughout the fermentation process. These beneficial bacteria can be commonly found in various fermented foods, including yogurt, sauerkraut, kimchi, and kefir [41]. By regulating the immune function of the host mucosa or by regulating the balance of intestinal flora, it can promote nutrient absorption and maintain intestinal health. The LAB that are widely encountered in fermented food include *Lactobacillus*, *Leuconostoc* and *Enterococcus*, *Weissella*, *Pediocossu*, etc. [40]. LAB are indeed involved in producing a wide range of fermented food products, including alcoholic drinks, fermented bread and noodles, fermented fish and meat, fermented dairy products, and fermented vegetables [42]. According to Sudhanshu et al. [43], *Lactobacillus plantarum* is commonly found in fermented vegetables due to the acid and salt resistance in the specific fermentation conditions. Kimchi contains *Leuconostoc*, *Lactobacillus*, and *Lactococcus*, which are responsible for the creation of unique sensory properties and nutritional properties [43]. Fish that has been fermented frequently contains *L. plantarum,* which has qualities that make it safe for consumption.

The presence of a live culture is dependent on the processing method and the specific food. An unsuitable process may affect the viability of the LAB. Commercial yogurt contains live cultures such as *Lactobacillus delbrueckii* subsp. bulgaricus that are intentionally added during production to create unique texture, flavor, and nutritional value. The survivability of probiotics in the gastrointestinal tract (GIT) is often regarded as critical for their potential health effects [44]. In the context of postbiotics, however, the vitality of probiotic bacteria may not be as important as the existence and activity of the bioactive compounds they create, such as organic acids, enzymes, peptides, polysaccharides, and other metabolites [45]. In the case of conventional probiotic treatments that ingest live cells, viability is critical for delivering the desired effects in the gastrointestinal system. According to Sahadeva et al. [46], the ability of the LAB to resist acid and bile is vital to indicate the survival rate of the bacteria in the intestinal transit and exert their potential benefits. As a result, some factors such as the types of strains and fermentation conditions need to be considered during fermentation [44].

Postbiotics can be characterized as metabolite byproducts produced by beneficial microorganisms throughout the growth and fermentation process that have a positive impact on a consumer’s health [47]. Numerous bioactive metabolites including organic acid (lactic acid, acetic acid), carbohydrates, enzymes, bacteriocins, vitamins (vitamin B12, riboflavin, and folate), and short-chain fatty acids are present in the postbiotics prepared from LAB [47,48]. The procedure strains of postbiotics can be naturally found in fermented food, which plays an important role in the production of bioactive metabolites, including those from bacterial (*Lactobacillus*, *Streptococcus*, and *Bifidobacterium*) and fungal species (*Saccharomyces*) [47]. The consumption of postbiotics may help in the enhancement of gut health, anti-inflammatory effects, and prevent respiratory infections.

### 1.4. Metabolism Characteristics of LAB

LAB have a number of vital metabolic characteristics that support their function in fermentation, including metabolizing sugar (glucose, lactose, and fructose) into lactic acid, bile tolerance, hydrolyzing protein, and antimicrobial properties [49]. Numerous beneficial compounds including organic acid, antibacterial, and exopolysaccharides are produced by metabolism. Lactic acid bacteria can indeed differ across distinct strains in terms of their specific metabolic characteristics and abilities. The genetic composition, growth conditions, external environment, and their adaptation to different environments determine the metabolism characteristics of LAB. For example, *Lactobacillus delbrueckii* subp. bulgaricus commonly used in yogurt production is associated with lactose metabolism, whereas the *Lactobacillus plantarum* found in fermented vegetables is able to metabolize a wide range of sugars [43]. Furthermore, the utilization of specific strains with known metabolic characteristics and improved control over the fermentation parameters are important for producing the desired quality of the product.

### 1.5. Product Synthesized by LAB

LAB are well known for their ability to decompose macromolecules in various food substances and synthesize lactic acid as the main product. Lactic acid is a significant bio-based compound that contributes to texture, flavor, and nutritional enhancement, and also reduces the pH value of the environment, which inhibits harmful substances. The product synthesized by lactic acid bacteria depends on the types of fermentation carried out [50]. On the other hand, these bacteria are also associated with the potential health attributed to the bioactive peptides, bacteriocins, vitamins, and exopolysaccharides [49].

LAB can yield byproducts that possess bioactivity and contribute to various health-promoting effects, including anti-allergic, modulate respiratory immunity, anti-gastric activity, anti-inflammatory, antimicrobial activity, and anti-oxidant effects [51]. EPS can be produced by several strains of LAB that have been demonstrated in numerous studies to lead to health modulation, such as anti-diabetic, cholesterol-lowering, anti-oxidant, anti-ulcer, and immunomodulatory properties [6]. Aside from these benefits, several strains have the ability to produce enzymes (proteases, lipases, and amylases) with various functionalities that increase nutrient absorption. LAB have been found to produce metabolites that exhibit antimicrobial properties. Finally, the organic acid (acetic acid and lactic acid) and bacteriocins produced by LAB exhibit anti-microbial activities.

#### 1.5.1. Organic Acids

Certain metabolisms, including sugar metabolism, can synthesize various types of organic acid, including lactic acid, acetic acid, butyric acid, and propionic acid, depending on the metabolic pathway. Lactic acid is the main product produced along the metabolic pathway, which is divided into L-lactic acid and D-lactic acid based on the different configurations around the chiral atom. The anaerobic condition throughout the glycolysis pathway results in the production of lactic acid which contributes to the sour flavor of fermented food, such as yogurt and pickles [52]. The fermentation can be divided into homo-lactic fermentation and hetero-lactic fermentation depending on the final product produced [26]. According to Thomas Bintsis [53], homo-lactic fermentation is the process in which lactic acid is the only type of acid, whereas hetero-lactic acid is involved in the production of lactic acid with other byproducts, such as carbon dioxide, ethanol, and acetic acid.

In the process of homo-lactic acid fermentation, glucose acts as the carbon source to create pyruvate through the glycolysis process, which is then subsequently converted to lactic acid by lactate dehydrogenase. The energy was previously generated in the form of NADH. As a result, only lactic acid is produced (one mole of glucose produces two moles of lactic acid and two ATP molecules) [54]. Lactobacillus and Lactococcus are examples of LAB during homo-lactic acid fermentation. Some homo-fermentative microbes can create formic acid under stressful conditions through mixed acid fermentation, including different carbon sources, pH values, or temperatures [55].

In contrast, hetero-lactic acid bacteria decompose the glucose into lactic acid alongside byproducts including acetic acid, ethanol, and carbon dioxide through the phosphoketolase pathway. Leuconostoc and Oenococcus are examples of hetero-lactic acid bacteria. Theoretically in hetero-lactic fermentation, one mole of lactic acid is created when one mole of glucose is decomposed [54]. Glucose 6-phosphate has been transformed into carbon dioxide, ribulose 5-phosphate, and NADPH via the pentose phosphate (PP) pathway [54]. Lactate dehydrogenase plays an important role in the production of lactic acid from pyruvate, and the configuration of the lactic acid is determined by its stereospecificity. L-lactase dehydrogenase is responsible for the synthesis of D-lactic acid, whereas D-lactase dehydrogenase is responsible for the synthesis of D-lactic acid [49]. Other than glucose, lactic acid bacteria can also metabolize fructose, mannose, or galactose. These hexoses serve as alternative carbon sources for the fermentation process [53].

**Figure 1:**
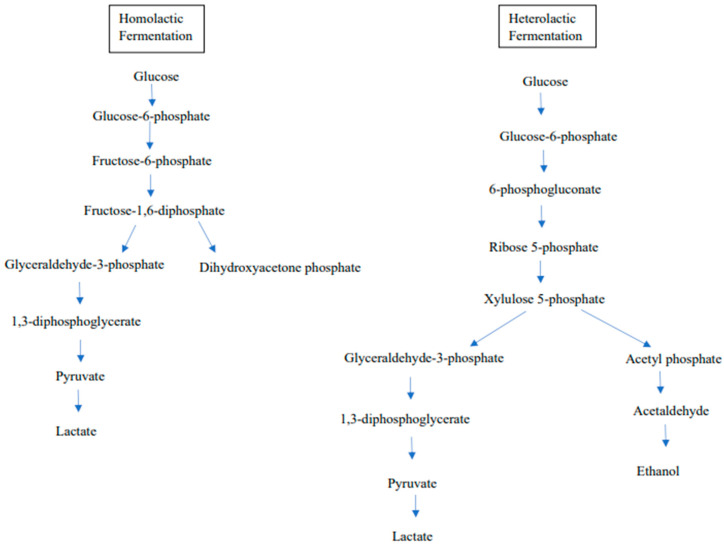
Homolactic fermentation and heterolactic fermentation in LAB [49].

The industrial production of organic acid can be performed by chemical synthesis and fermentation methods for commercial applications. Numerous studies have been carried out by the food industry to improve the purity of lactic acid, as it is important in terms of safety, product stability, flavor, and aroma. Saccharification and fermentation (SF) and separate hydrolysis and fermentation (SHF) are commonly applied in the food industry in order to produce lactic acid with high optical purity and to reduce sugar residue [56]. *Lactobacillus*, *Leuconostoc*, and *Streptococcus* are known to produce various organic acids as end products to prevent the spoilage of food and to improve the taste [57]. Apart from as a flavor enhancer, organic acid in food can be utilized as a food preservation, cleaning, and sanitizing agent due to its antimicrobial and antioxidant properties [57]. Although the LAB mainly produces lactic acid, it can also produce 3-hydroxy propionate, acetate, and succinate. For instance, *Limosilactobacillus reuteri* are capable of producing 3-hydroxypropionic acid as a metabolic byproduct of glycerol metabolism and *Lactiplantibacillus pentosus* can produce acetic acid [49]. The metabolic capacity of LAB to generate organic acid plays a significant role in their probiotic functionality. Figure 1 has shown homolactic fermentation and heterolactic fermentation in LAB.

#### 1.5.2. Bacteriocins

Bacteriocins are antimicrobial peptides or proteins produced by both Gram-positive and Gram-negative bacteria against different closely related bacteria [58]. Lactic acid bacteria have been extensively documented by several studies for their probiotic properties, mycotoxin degradation, and inhibition of pathogenic bacteria [59]. According to Kumariya et al. [60], the bacteriocin function comprises the target bacteria’s cell integrity, impedes cellular processes, and interferes with the synthesis of DNA or protein. Various environmental factors, including pH, incubation temperature, nutritional availability, and composition in the growth medium, have a significant impact on bacteriocin synthesis.

Bacteriocins can be divided into four different classes based on their biochemical and genetic characteristics. Class I bacteriocins, also known as lantibiotics, are small post-translationally modified peptides (<5 kDa) that are characterized based on the presence of lanthionine and methyllanthionine [26]. Nisin produced by *Lactococcus lactis* is indeed one of the well-known examples of Class I bacteriocin that have been extensively studied [61]. According to Svetoslav D. Todorov [59], Class II is the non-lantibiotic, which can be divided into four subclasses depending on their characteristics: Class II a (listeria-active bacteriocins), Class II b (two-peptide complexes), Class II c (the sec-dependent bacteriocins), and Class II d (unclassified small heat-stable non-lathionine bacteriocins). This bacteriocin is small with an amphiphilic helical structure (<10 kDa) that causes cell death by disrupting the integrity of the cell [62]. Class III bacteriocins are the large bacteriocins (>30 kDa) that are synthesized by the *Lactobacillus helveticus*. Bacteriocins generated by bacteria typically need to be secreted from the cell in order to interact with target cells and exhibit their antimicrobial effect [49].

The inherent characteristics of LAB bacteriocins confirm their potential for application in the food industry. Bacteriocins have been extensively used in food preservation, and their potential for use in cancer therapy and oral care [63] is well known as a natural food preservative that is secreted by *Lactococcus lactis* and works against the Listeria monocytogenes [49]. This natural preservative is commonly used in the dairy industry and canned food industry for its antimicrobial properties, improvement of sensory properties, and food quality. For instance, nisin has been reported to inhibit the growth of Gram-positive bacteria, including *Lactilactobacillus sakei* in ham production [64]. In addition, *Lactiplantibacillus plantarum* can prevent and extend the shelf life of raw minced beef by inhibiting the growth of spoilage microorganisms [65].

#### 1.5.3. Vitamins

The metabolites and enzymes produced during the fermentation process can contribute to the bioavailability and production of several vitamins, including vitamin B12, vitamin C, riboflavin (B2), and folate [49]. The capability of the LAB in the synthesis of various vitamins is dependent on the strains and species. According to Zhen Wu et al. [66], *L. plantarum* showed the highest folic acid production compared to other LAB. Moreover, *Lactococcus lactis* and *Streptococcus thermophilus* are common LAB that are used as the starter culture in yogurt production due to their folate synthesis capabilities [67].

Foods that contribute to the bioavailability and synthesis of vitamins during fermentation might be regarded as fortified foods, which are significant to a particular demographic. Folate is a water-soluble vitamin that is essential in the biosynthesis of nucleotides and proteins, including DNA replication [67]. The folate is synthesized from para-aminobenzoic acid (PABA) through a series of reactions. The Lactobacillus strains require the presence of the PABA in the culture medium synthesis of the folate. Several studies have shown that the capability to synthesize folate is dependent on the species, strain, and culture conditions [68]. The development of non-folate-producing LAB is determined by the amount of folate present in the medium, as this strain may need an exogenous source for growth, whereas folate-producing LAB can regulate folate biosynthesis. It can synthesize folate when the medium is deficient in it [69]. Most LAB, especially *Streptococcus* and *Lactobacillus*, are examples that have the ability to synthesize folate [70].

Riboflavin, also known as vitamin B2, is a water-soluble vitamin that serves as the precursor of the flavin adenine dinucleotide (FAD) and flavin mononucleotide (FMN), which are essential for the coenzymes in the redox reactions within the cell [71]. The genes encoding riboflavin synthase in LAB are clustered on the rib operon and contain the genes responsible for the synthesis of riboflavin. The guanosine triphosphate and 5-phosphate ribose are converted into riboflavin, catalyzed by the products of riboflavin synthase genes, namely RibC, RibB, RibA, and RibH. Vitamin synthesis during the fermentation process can be considered as the nutritional fortification of the food. LAB can enhance the nutritional content of fermented food by producing vitamin K and vitamin B12, which contribute to functional food. For example, *S. thermophilus*, used as a starter culture in the dairy product industry, can synthesize folate. Moreover, *Lactococcus laudensis* and *Lactococcus hircilactis* are added to fermented milk production to produce folate that enhances the nutritional value of the product [72].

#### 1.5.4. Exopolysaccharides (EPS)

Exopolysaccharides are biodegradable polymers formed from sugar monosaccharides, which are synthesized and secreted by LAB into their surrounding environment [73] (Pinar Sanibaba, 2016). EPS is important in order to provide the specific texture, viscosity, and probiotic properties of fermented food. These polymers are widely used as stabilizers and emulsifying agents in the food industry due to their water-holding capacity [74]. On the other hand, EPS have been associated with the potential health benefits of existing anti-inflammatory activities, and antitumor and anticancer properties [75]. Several studies have shown that EPS contribute to gut health and promote bacterial colonization by forming a protective matrix [76]; *Lactiplantibacillus plantarum*, *Fructilactobacillus*, *Lactococcus*, *Weissella*, and *Leuconostoc* are especially capable of producing different kinds of EPS based on the strain [77,78].

These polymers can be classified into homopolysaccharides (HoPS) and heteropolysaccharides (HePS) based on the composition of the sugar unit. HoPS are polysaccharides composed of a single type of monosaccharides, whereas HePS consist of different types of monosaccharides [79]. The sugar composition and chain length of the EPS depend on the species of LAB that contribute to the wide range of applications in the food industry [80]. The biosynthesis of HoPS is considered to be a simple process compared to the other polysaccharides syntheses, as it does not involve the active transportation stage in the synthetic pathway. These polymers are synthesized by glycansucrases and fructansucrase, respectively, by allowing the glucose and fructose to act as the glycosyl donors in this synthesis [73]. In contrast, HePS biosynthesis is more complex due to the sugar composition, molecular weight, and linking pattern. It is involved in the sugar activation of the sugar nucleotide precursor formation, polymer chain elongation, branching, and the export of the EPS [49,81,82]. Environmental factors, including pH, temperature, time, and also the strain of the LAB, influence EPS production. For example, Xue Han et al. [83] showed that the combination of *Streptococcus thermophilus* and *Lactobacillus delbrueckii* subsp.bulgaricus produce higher EPS content and better sensory texture of yogurt.

EPS can be considered to be valuable additives, including thickeners, fat substitutes, and texturizers that improve the rheological properties, sensory attributes, and texture of fermented food. EPS include glucans, used as the stabilizer, thickener, and emulsifier in food production to improve the texture and consistency of products. EPS-producing starter culture can be utilized in the production of fermented food to improve the rheological properties of the product. Adding the EPS-producing strain of *Lactobacillus plantarum* improves the texture properties, sensory value, and moisture content of low-fat cheddar cheese [49]. Moreover, *Lactococcus lactic* F-mou synthesis of the EPS shows excellent water-holding capacities, antioxidant properties, and inhibitory effects against pathogenic bacteria [84].

#### 1.5.5. Gamma-Aminobutyric Acid

Gamma-aminobutyric acid (GABA) is a neurotransmitter catalyzed by glutamate decarboxylase (GAD) and pyridoxal-5′-phosphate [49]. This substance is regarded as one of the bioactive compounds created by LAB that may be beneficial to the consumer’s health. GABA can enhance the metabolism of the brain cells that regulate the growth of hormone secretion, protein synthesis, fat burning, and blood pressure by improving oxygen delivery and blood flow [85]. The potential health effects of GABA include antidepression by promoting relaxation and reducing anxiety, lowering cholesterol, blood pressure regulation, and anticarcinogenic properties. *Lactobacillus namurensis*, *Lactobacillus paracasei*, and *Lactobacillus brevis* are examples of LAB that have demonstrated the capacity to produce GABA due to the presence of GAD [85]. Moreover, some *Streptococcus thermophilus*, *Lactococcus lactis*, and *Leuconostoc* strains have recently been found to be able to produce GABA [86].

The primary mechanism of intracellular GABA production is the L-Glu decarboxylation process. The decarboxylation reaction of L-glutamate to produce aminobutyric acid is carried out by GAD and pyridoxal-5′-phosphate (cofactor). There have been a number of significant genes identified that control the production of gamma-aminobutyric acid (GABA). According to Chang Jiang Lyu [87], the mutation of the GadA gene in *Levilactobacillus brevis* makes it easier for L-monosodium glutamate (MSG) to be converted to GABA. The inhibition of the GABA aminotransferase showed an increase in GABA production [86]. Additionally, the generation of GABA is influenced by several factors, including temperature, pH, culture composition, and time [88]. The addition of glutamate in the medium shows the increasing concentration of GABA by *L. paracasei* and *L. brevius.* Some microorganisms with a high level of safety that are able to produce GABA can be added to the food to act as fortification products. Additionally, LAB can ferment cheese, yogurt, and milk to act as GABA-enriched goods [89]. Currently, *Levi Lactobacillus brevis* is typically utilized in fermentation to produce GABA, as it is able to convert monosodium glutamate and L-glutamic acid into GABA.

#### 1.5.6. Flavor Substances

In addition to the possible health benefits, fermented food is known for its distinctive flavor. The presence of desirable flavor compounds is the key factor in determining the sensory characteristics of fermented food. According to Coolbear et al. [90], organic acids, alcohols, ketones, and esters are some of the flavoring compounds made by lactic acid bacteria. LAB can function either as the dominant bacteria or combine with other bacteria to produce flavor substances. Generally, the flavor substances are generated by biosynthesis, the enzymatic reaction by the enzyme inside the food, oxidative decomposition by the exposure of heat with oxygen, and the pyrolysis process where the organic compound decomposes because of high temperature [91]. During yogurt production, the flavor substances can be generated by amino acids, fatty acids, and carbohydrates. According to Chen Chen et al. [52], *Lactococcus lactis*, *Lactobacillus species*, and *Streptococcus thermophilus* are responsible for the production of flavor substances, including alcohol and esters. In addition, the addition of LAB in sourdough fermentation contributes to the sour aroma [49]. There are multiple metabolic pathways involved in the synthesis of flavor substances. The citric acid pathway, also known as the Krebs cycle, is one of the metabolic pathways that synthesize intermediate compounds such as citric acid and succinic acid, which then contribute to flavor formation [49]. In addition, sugar metabolism leads to the production of sugar alcohol, which contributes to the sweet taste of the food.

### 1.6. Application of LAB in Clinical Nutrition

LAB have been used in clinical nutrition for a range of purposes and are well known for their health-promoting qualities. LAB also possess therapeutic properties that are important to enhance human health. Because they have been demonstrated to enhance immune function, promote gut health, and lower the risk of infections, LAB are frequently used as probiotics. To increase nutrient absorption and enhance gut health, LAB are also utilized in enteral and parenteral nutrition [7]. Additionally, it has been established that LAB have anti-inflammatory and antioxidant properties, making them a possible therapeutic choice for a number of conditions, such as inflammatory bowel disease, irritable bowel syndrome, and specific types of cancer.

#### 1.6.1. LAB in the Management of Lactose Intolerance

The symptoms of lactose intolerance, an inherited autosomal recessive trait with incomplete penetrance, are caused by the non-absorbed lactose in the small intestine moving to the colon, where it is metabolized by the intestinal flora and produces short-chain fatty acids and gas, primarily hydrogen (H_2_), carbon dioxide (CO_2_), and methane (CH_4_). Lactose intolerance symptoms vary depending on the residual lactase activity and can cause severe digestive disorders. Colonic adaptation of probiotics is one of the treatments for lactose intolerance [92]. In probiotic preparations, the most common organisms include *Lactobacillus*, *Escherichia*, *Bifidobacterium*, *Bacillus*, *Enterococcus*, *Streptococcus*, and some fungal *Saccharomyces* strains. Cano-Contreras et al. [93] highlighted the efficacy of probiotics in reducing lactose intolerance symptoms. It was suggested that the probiotics help in modifying the pH of the intestine. Some strains of LAB also help in the secretion of bacteria lactase into digestive systems [94].

The administration of probiotic supplementation increases the concentration of β-galactosidase, which helps to alleviate the symptoms of lactose malabsorption. A previous study found the effect of *L. bulgaricus* strains increases the amount of β-galactosidase [95]. Pakdaman et al. [96] demonstrated the effectiveness of LAB in reducing the symptoms of lactose intolerance, whilst Roškar et al. [97] reported a non-significant difference between the placebo group and the probiotic group in reducing lactose intolerance symptoms, particularly diarrhea and flatulence, as compared to the baseline. Nevertheless, this study found an improvement in alleviating the symptoms after LAB consumption. A recent meta-analysis reported the effectiveness of probiotic administration in alleviating lactose intolerance symptoms among adults [98] (Ahn et al. 2023).

#### 1.6.2. LAB in the Treatment of Diarrhea

For decades, malnutrition, particularly undernutrition in hospitalized patients, has received significant attention [99]. Critically ill patients frequently experience non-contagious diarrhea, which has been linked to hospital stay. Antibiotic-associated diarrhea is very common among critically ill patients and it has been shown that microbes are not the major source or risk factor of non-infectious diarrhea [7]. A significant number of microorganisms that are part of the gut microbiota, a complex ecosystem, play important roles in the growth, metabolism, and aging of the host. The composition and phenotype of intestinal microorganisms significantly change during critical illness and the subsequent medical interventions, making the patient more vulnerable to opportunistic infections, even developing System Inflammatory Reaction Syndrome (SIRS) or Multiple Organ Dysfunction Syndrome (MODS) [100]. A wide range of antibiotics are used, which result in the loss of beneficial bacteria from the gut. Diarrhea is a major clinical adverse effect that leads to poor prognoses, such as poor wound healing, electrolyte imbalance, the loss of fluid, hemodynamic instability, and a deficiency of nutrients.

Many beneficial bacteria have been isolated and used to treat gastrointestinal symptoms. LAB have the potential to improve gut health by producing lactic acid, bacteriocins, and short-chain fatty acids, all of which serve to keep the balance of gut microbiota and prevent the overgrowth of harmful bacteria. Probiotics work by inhibiting the action of pathogenic bacteria, aiding immunomodulation, enhancing gut barrier function, and assisting in the release of neurotransmitters. Thus, probiotics aid in the maintenance of a sound gut-brain axis [101,102]

Bacteroidetes and Firmicutes phyla probiotics, including *Lactobacillus*, *Bifidobacterium*, and *Streptococcus salivarius* subsp., have been used to treat a range of intestinal symptoms, including diarrhea-dominant irritable bowel syndrome (IBS), diarrhea, inflammatory bowel disease, and antibiotic-induced diarrhea. A recent systematic review and meta-analysis included studies (all conducted in China) that showed that probiotic significantly reduces gastrointestinal complications in severe stroke patients, according to a new systematic review and meta-analysis of studies (only conducted in China) *p* < 0.0001 [103]. Skrzydło-Radomańska et al. [104] reported that the use of multi-strain synbiotic preparations was associated with a significant improvement in symptoms of IBS. A pilot randomized study also revealed the effectiveness of sporulated bacillus in alleviating the symptoms of diarrhea among patients on enteral nutrition compared to fiber-enriched formula alone [105].

Two recent meta-analyses reported different findings. A meta-analysis study by Alsuwaylihi and McCullough [7] found a potential effect of probiotics (*Lactobacillus rhamnosus* GG and *Bacillus cereus* on clinical or diarrheal outcomes in critically ill patients. Lee et al. [106], in another meta-analysis study, did not support the beneficial effect of probiotics on the treatment of diarrhea in critically ill patients. As a result, the optimum dosage and effectiveness of probiotics on the reduction of diarrhea remains inconclusive.

#### 1.6.3. Immunomodulatory Effects of LAB

Utilizing LAB in enteral nutrition has drawn more attention in recent years, especially in critically ill patients who receive nutrition through a feeding tube. It is also worth noting that the use of LAB in enteral nutrition should be closely monitored and tailored to each patient’s medical history, health state, and other variables. In vivo evidence of probiotics’ ability to suppress the generation of proinflammatory cytokines and stimulate IgA secretion has been documented in several investigations in recent years [107]. The gastrointestinal tract is an essential microbiologically active ecosystem that plays a crucial role in the working of the mucosal immune system.

LAB, including *Lactobacillus* and *Streptococcus lactis*, have shown a positive effect in terms of improving the immunity of individuals. Wei et al. [108] explored the clinical effect of compound LAB capsules with Escitalopram (a medicine used to treat depression) on small intestinal bacterial overgrowth (SIBO) in patients with depression and diabetes. CD^3+^ and CD^+4^ showed a greater increment among individuals supplemented with LAB compared to the control. *Lactobacillus* and *Streptococcus lactis* act on the body to multiply in the intestinal tract, increase lactic acid production, and inhibit the reproduction of spoilage bacteria. It was found that the modulation of the immune system by gut microbiota is via the production of molecules with immunomodulatory and anti-inflammatory functions that can stimulate immune cells. Immunomodulatory effects were produced by the probiotic interaction with epithelial cells and dendritic cells, as well as with monocytes/macrophages and lymphocytes [109].

LABs were found to synthesize low molecular weight compounds such as organic acid and large molecular weight antimicrobial compounds (known as Bacteriocins). Bacteriocins produced by LAB probiotics exhibit strong inhibitory effects against pathogenic Gram-negative bacteria, such as H. pylori. Oral administration of LAB increases Paneth cells based in the small intestine [110]. Aggravations or alterations of the normal intestinal microflora in the gastrointestinal gut are the common cause of inflammatory bowel diseases such as Crohn’s disease. Additional probiotics in individuals’ diets have been shown to replenish or modify gut microflora [111].

Ventilator-associated pneumonia (VAP) is the most prevalent fatal complication of nosocomial infection (NI) in intensive care unit (ICU) patients. Beneficial bacteria play an important role in maintaining the intestinal barrier and host immunity. In a meta-analysis, Batra et al. [112] found that probiotics supplementation decreased the incidence of VAP, the length of mechanical ventilation, the length of ICU stays, and in-hospital mortality among ventilated critically sick ICU patients. A previous study highlighted the supplementation of two capsules of probiotic containing LAB had a lower incidence of statistically microbiologically confirmed VAP [113]. Despite high-quality random trials on the efficacy of LAB in preventing VAP, this dietary therapy remains highly controversial in the reduction of VAP among patients with trauma or other critical illness [114].

The disease known as sepsis, on the other hand, can arise as a side effect of an infection and is potentially fatal. It happens when the body’s reaction to an infection is thrown off balance, resulting in a systemic inflammatory response across the body. Numerous studies have shown that nutritional therapy for malnourished individuals reduces the chances of infection complications, wound inflammation, and mortality [115]. In critically ill patients, the commensal microbiota deteriorates, in which most ICU patients are associated with infections and mortality [116]. Sepsis among critically ill patients has been associated with lowering microbiota in the gut. The integrity of the intestinal epithelial barrier and the absorptive function of the intestinal mucosa may be compromised as a result of changes in the intestinal microbial composition during severe illness. LAB has anti-inflammatory properties and may aid in reducing the risk of infection and sepsis in critically ill patients [117]. The isolated bacteria are also termed “probiotics”. The administration of synbiotics (probiotic and prebiotics containing lactic acid bacteria) containing the *B. breve* strain and the *L. casei* strain, in an amount of 3 g per day, was initiated within 3 days after admission through enteral feeding. The synbiotic is used to inhibit pathogenic bacteria and toxins through signal interaction and prevent septic complications. In this study, the numbers of *Bifidobacterium* sp. and total *Lactobacillus* sp. in the synbiotic group also showed an increment [118]. Shimizu et al. [118] reported that the use of the synbiotics of LAB had fewer complications of diarrhea and ventilator-associated diarrhea. The usage of probiotics resulted in a better outcome in terms of lowering overall ICU infection rates, particularly VAP [7].

#### 1.6.4. LAB and Hepatoprotective Effects

Ethanol exposure is strongly linked to alcoholic liver disease (ALD), a chronic illness with the highest incidence and mortality rate in the world [119]. ALD includes alcoholic fatty liver, alcoholic steatohepatitis, alcoholic hepatitis, alcoholic fibrosis, alcoholic cirrhosis, and alcoholic hepatocellular carcinoma. In recent years, studies have found a close relationship between alcohol and gut microbiota [120]. Alcohol can increase intestinal permeability, which leads to liver damage with the release of reactive oxygen species (ROS), adhesion molecules, chemokines, and proinflammatory cytokines; therefore, the use of probiotics may limit the progression of ALD by changing the intestinal bacteria [19].

Bakhshimoghaddam et al. [121] reported a reduction in the liver function test profile including the serum concentration of alanine aminotransferase, aspartate aminotransferase, alkaline phosphatase, and γ-glutamyltransferase among non-alcoholic fatty liver disease (NAFLD) patients receiving supplementations of *Bifidobacterium animalis* compared to the control group. Nevertheless, Mohamad Nor et al. [122] found no significant effect of combining LAB containing *Lactobacillus* and *Bifidobacterium* on the liver function test profile. Variceal bleeding has a high incidence among patients with liver cirrhosis and leads to a high risk of mortality and morbidity.

#### 1.6.5. LAB for Prevention and as a Potential Natural Anti-tumour Drug

Exopolysaccharides produced by lactic acid bacteria, as one of the most important functional components of LAB metabolic products, have attracted considerable attention in recent years due to their unique physicochemical properties [123] and their ability to modulate cancer cell proliferation and apoptosis both in vitro and in vivo [124]. The effectiveness of LAB in clinical trials has been limited and inconclusive. More clinical trials are necessary to establish the potential benefits of LAB in the prevention and treatment of cancer. In a study by Zhao et al. [125], probiotic-enriched nutrition formula among gastric cancer patients who received enteral nutrition had a lower number of surgery side effects, such as diarrhea and intestinal disorder, compared to those with fiber-free or fiber-enriched nutrition formula.

Recent studies have indicated the beneficial role of probiotics in the prevention of carcinogenesis and have presented new promising therapeutic options. However, the safety used for cancer patients remains inconclusive [126]. More research is needed to conclude the potential benefits of probiotics for cancer patients.

#### 1.6.6. LAB in the Management of Glycemic Control

Diabetes remains an overwhelming health problem worldwide despite advancements in healthcare management. Probiotic supplements do not cause clinically significant decreases in Hemoglobin A1c (HbA1c) levels in people with type 2 diabetes, but they do cause marginally clinically significant reductions in fasting glucose and fasting insulin levels. Multi-strain and high-dose probiotics have had a larger positive impact on glucose homeostasis compared to single-strain and low-dose probiotics. Probiotic therapy may also be more successful in people who are older and have a high baseline Body Mass Index (BMI) [127]. The supplementation of 10^8^ CFU of *L. casei* 01 for 8 weeks significantly reduced the serum fetuin-A level, fasting blood sugar, insulin concentration, and insulin resistance [128]. In contrast, the supplementation of probiotic yogurt containing *Lactobacillus acidophilus* and *Bifidobacterium lactis* showed no significant effect on fasting blood glucose, whilst there was a reduction in hemoglobin A1c compared to the placebo group [129]. This discrepancy might be due to the period of study, the dosage, and the use of LAB. Further study is needed with longer interventions to better conclude the effectiveness of LAB on blood glucose control. Table 2 has summaries the functional properties of LAB in the management of nutrition.

### 1.7. Challenge of Lactic Acid Bacteria as a Food Nutrient

While lactic acid bacteria have shown promise in the food industry, their use as food nutrients in clinical nutrition poses additional challenges. Not all clinical trials have shown improvement in the health of individuals receiving probiotic medication, and very few have indicated that probiotic strains may be the causal agents of opportunistic illnesses. These very uncommon illnesses are mostly seen in higher-risk categories, such as immunocompromised people. In immunocompromised individuals, there is a chance that specific LAB strains might increase their risk of infection or sepsis, among other safety issues. It is also important to ensure the LAB used as dietary nutrients do not compromise the efficiency or absorption of other drugs [133]. Future studies should emphasize the drug-nutrient relationship in the creation and delivery of the therapeutic effects of LAB. More investigations into the probiotic-drug and probiotic-gut microbiota interactions are required in the near future because the precise mechanisms are still partially understood [134]. Additionally, LABs are known to have immune-modulating effects on the host, making them a prospective therapeutic and preventative choice for a number of illnesses, including inflammatory disease. Understanding the genus and species of the probiotics is crucial to attaining the desired effects on the host, since probiotic effects vary depending on the dose, circumstance, and strain [135]. LAB usage can be considered generally safe for healthy individuals. There is an urgent need for further evidence on adverse events, particularly in immunocompromised hosts and vulnerable groups in both the short and long term [136].

## 2. Conclusions

Understanding the characteristics of LAB and their application in the management of nutrition is important for ensuring optimal health outcomes. These nonpathogenic bacteria are useful in technological and industrial processes. LAB is characterized as a phylogenetic and functionally diverse taxonomic order of bacteria. By modulating the gut microbiota, LAB supports better digestion, increase nutritional absorption, improve antimicrobial properties, and boost immunological function. The safety profiles of various LAB as a function of various genera, species, and strains, as well as their applicability in a variety of people or populations at risk, have gained substantial interest. The use of LAB to provide health benefits to the host requires the specification of the dosage regimens and the duration of use as recommended by the manufacturer of each individual strain or product based on scientific evidence, and as permitted in the country of sale, as per the Joint FAO/WHO (2002) guidelines on LAB. The minimal daily dose required for any LAB-containing product to bestow a particular health benefit or advantage should also be specified. Clear proof of this goal should come from in vitro, animal, or human clinical investigations, if feasible.

## Figures and Tables

**Table 2:** Functional properties of LAB in the management of nutrition.

Therapeutic Effects	Lactic Acid Bacteria (LAB) Strain	Remarks	References
Lactose intolerance	*Lactobacillus acidophilus*,	Method: Supplementation *L. acidophilus* Results: Abdominal symptom (LAB < control)	[96]
	*B. animalis*, *Lactobacillus plantarum*	Methods: Supplementation of *B. animalis* Results: Abdominal symptoms (no significant difference)	[84]
	*Lactobacillus plantarum*, *P. acidilactici*	Method: Supplementation of *Lactobacillus plantarum* and *P. acidilactici* among lactose intolerance patients Results: Total symptom score of lactose intolerance (LAB < control)	[93]
Gastrointestinal problem: diarrhea	*Baccilus cereus*	Method: Supplementation of 20 mL/day *Baccilus cereus* or soluble fiber (control) among patients with diarrhea on enteral feeding Results: Ceasing the diarrhea incident (no significant difference), duration to stop diarrhea (*B. Cerius* group < control)	[105]
	*Lactobacillus rhamnosus*, *Lactobacillus acidophilus*, *Bifidobacterium lactis*, *Bifidobacterium longum*, *Bifidobacterium bifidum*	Method: Synbiotics supplementation among diarrhea-dominant IBS for 8 weeks Results: After intervention, feeling of incomplete bowel movements, flatulence, pain, stool pressure, and diarrheal stools (synbiotics group < control)	[104]
Immunomodulatory effect	*Bifidobacterium breve*, *Lactobacillus casei*	Method: 3 g supplementation of synbiotics (*Bifidobacterium breve* and *Lactobacillus casei*) within 3 days after admission Results: Enteritis and penumonia incidence lowered in synbiotics group compared to control	[118]
	*Lactobacillus and Streptococcus lactis*	Method: Lactic acid bacteria capsule among depression and diabetes patient Result: Reduction of self-rating anxiety scale, IL-2 and TNF-*α*, fasting plasma (LAB > control), and increment of CD^+4^ (LAB > control) Adverse effect LAB < control)	[108]
	*Lactobacillus casei*, *Lactobacillus acidophilus*, *Lactobacillus rhamnosus*, *Lactobacillus bulgaricus*, *Bifidobacterium breve*, *Bifidobacterium longum*, *Streptococcus thermophiles*	Methods: Supplementation of 1 capsule/12 h among VAP multi-trauma patients Results: VAP (intervention group < control)	[130]
	*Lactobacillus rhamnosus*	Method: Supplementation of 2 × 10^9^ Colony Forming Units (CFU) of *Lactobacillus rhamnosus GG* on a twice daily basis among ventilated medical ICU patients Results: VAP (no significant difference between LAB and the control)	[131]
	*L rhamnosus GG*	Method: Enteral *L rhamnosus GG* twice daily among patients on ventilation Results: VAP incidence (no significant difference between both the intervention group and the control)	[132]
Hepatoprotective effect	*Bifidobacterium animalis*	Method: Supplementation of 300 g synbiotics yogurt (B. animalis and inulin) or conventional (control) among NAFLD patients Results: Grades of NAFLD (synbiotics group < control), reduction in serum concentration of alanine aminotransferase, aspartate aminotransferase, alkaline phosphatase, and γ-glutamyltransferase (synbiotics group > control)	[121]
	*Lactobacillus*, *Bifidobacterium*	Methods: Supplementation of probiotics sachet or placebo for 6 months among NAFLD patients Results: No significant difference in LiverFAST analysis (steatosis, fibrosis, and inflammation scores), alanine aminotransferase	[122]
Treatment of cancer	*Bifidobacterium*, *lactobacillus*	Method: Gastric cancer patient receiving fiber-free nutrition formula (FF group), fiber-enriched nutrition formula (FE group), and fiber- and probiotic-enriched nutrition formula (FEp group) Results: The FEP group had the lowest number of diarrhea and intestinal disorders. No significant difference in the lymphocyte count, albumin, prealbumin, and transferrin levels	[125]
	*Bifidobacteria*, *Lactobacillus*	Method: Supplementation of probiotics + glucose solution or glucose solution (control) among colorectal cancer patients undergoing radical resection Results: Increase in intestinal micro-ecological environment and strengthening of the intestinal mucosal barrier function (glucose solution + probiotic group > glucose group), duration of early recovery of inflammatory response (glucose solution + probiotic group > glucose group)	[120]
Glycemic control	*Lactobacillus casei*	Method: 10^8^ CFU of *L. casei* supplementation for 8 weeks among type 2 diabetes mellitus Result: Serum fetuin-A level, fasting blood sugar, insulin concentration, and insulin resistance significantly decrease among *L. casei* supplementation compared to the control	[128]
	*Lactobacillus acidophilus*, *Bifidobacterium lactis*	Methods: 200 g/d yogurt containing probiotic 4.65 × 10^6^ CFU/g or placebo group received 200 g/d conventional yogurt Results: No significant different in fasting plasma glucose (FPG), hemoglobin A1c (HbA1c)	[129]

## Data Availability

Not applicable.
